
JOURNAL INFORMATION
==============================
NLM Title Abbreviation: Hemasphere
Journal ID: Hemasphere
NLM Journal ID: 101740619
Journal ID: HEM3

HemaSphere

EISSN: 2572-9241
Publisher: Wiley

ARTICLE INFORMATION
==============================
PMCID: PMC12856515
DOI: 10.1002/hem3.70323
Article ID: HEM370323
Article version: 1
Subjects: Issue Information, Issue Information

Issue Information

Electronic publication date: 2026 Jan 30
Publication date: 2026 Feb
Volume: 10
Issue: 2
Electronic Location ID: e70323
Copyright: © 2026 The Author(s). HemaSphere published by John Wiley & Sons Ltd. on behalf of European Hematology Association.
License: This is an open access article under the terms of the http://creativecommons.org/licenses/by/4.0/ License, which permits use, distribution and reproduction in any medium, provided the original work is properly cited.
License URL: https://creativecommons.org/licenses/by/4.0/

Figure count: 0
Table count: 0
Page count: 4

source-schema-version-number: 2.0
cover-date: February 2026
details-of-publishers-convertor: Converter:WILEY_ML3GV2_TO_JATSPMC version:6.6.8 mode:remove_FC converted:30.01.2026

==============================

### HemaSphere

Vol. 10 - Issue 2

February 2026

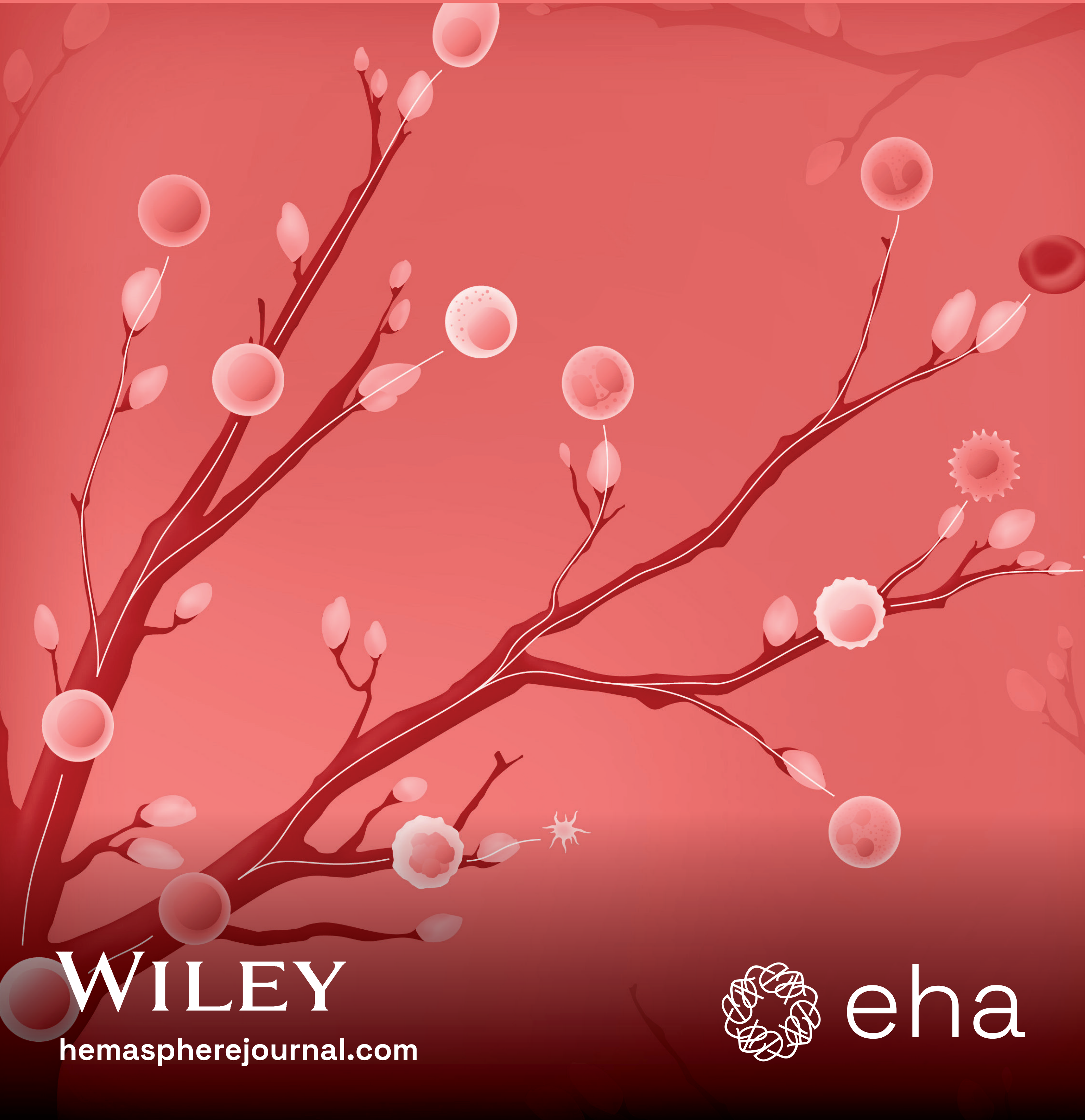

WILEY

hemaspherejournal.com eha

### Aims and Scope

*HemaSphere* publishes results of highly relevant basic, translational, and clinical research in hematology. We are especially looking for strong studies reporting novel findings that are of high impact to the field of hematology.

Review articles and guideline articles provide clear overviews and discussions of new developments as well as recommendations for patient care.

*HemaSphere* is the premier hematology information resource, which is enhanced by the HemaTopics and HemaPolicy sections of the journal. These sections provide insightful discussions on all aspects related to hematology, including summaries of important articles, reports of new therapies, discussions on European policy, and other hematology news items. *HemaSphere* is guided by Editor in Chief Jan Cools and Deputy Editor in Chief Claire Harrison. The Editors and Editorial Board of *HemaSphere* are international research and clinical leaders, representing a diverse range of areas of hematologic expertise.

### Open Access Licensing and Copyright

*HemaSphere* is an Open Access journal: authors of accepted articles pay an article publication charge, and their articles are published under a Creative Commons license.

Certain funders mandate a particular type of CC license is used. All other authors can choose between the CC BY-NC and CC BY-NC-ND Creative Commons Licenses.

For more information about the licenses offered by this journal, please visit Wiley Author Services.

### Disclaimer

The Publisher, European Hematology Association, and Editors cannot be held responsible for any errors in or any consequences arising from the use of information contained in this journal. The views and opinions expressed do not necessarily reflect those of the Publisher, European Hematology Association, or Editors, neither does the publication of advertisements constitute any endorsement by the Publisher, European Hematology Association, Editors, or Authors of the products advertised. Wiley Open Access articles posted to repositories or websites are without warranty from Wiley of any kind, either express or implied, including, but not limited to, warranties of merchantability, fitness for a particular purpose, or non-infringement. To the fullest extent permitted by law Wiley disclaims all liability for any loss or damage arising out of, or in connection with, the use of or inability to use the content.

HemaSphere eha

### Editorial Board

### Editor in Chief
**Professor Jan Cools**
*VIB-KU Leuven Center for Cancer Biology, Leuven, Belgium*

### Deputy Editor in Chief
**Professor Claire Harrison**
*Guy's and St Thomas' NHS Foundation Trust, London, United Kingdom*

### Associate Editors
Simone Ferrero, *University of Torino, Italy*
Adele Fielding, *University of York, Hull York Medical School, York, United Kingdom*
Simon Hallam, *St. Bartholomew's Hospital, Barts Health NHS Trust, London, United Kingdom*
Steffen Koschmieder, *University Medical Center RWTH, Aachen, Germany*
Riitta Lassila, *University of Helsinki, Helsinki, Finland*
Marek Mraz, *CEITEC Masaryk University and University Hospital Brno, Czech Republic*
Paula Rodríguez Otero, *Clínica Universidad de Navarra, Spain*
Jürg Schwaller, *University Hospital Basel, Switzerland*
Francesca Vinchi, *Emory University School of Medicine, Atlanta, Georgia, United States*

### Scientific Editors
Charles de Bock, *Children's Cancer Institute, Sydney, Australia*
Stephen Hibbs, *Queen Mary University of London, United Kingdom*
Mandy Lauw, *Erasmus University MC, Rotterdam, The Netherlands*
Lauren Merz, *University of Michigan, Ann Arbor, Michigan, United States*
Edeghonghon Olayemi, *Vancouver Coastal Health (Richmond Hospital Site), Richmond, British Columbia, Canada*

### Statistics Editor
Robert Hills, *University of Oxford, United Kingdom*

### Editorial Manager, European Hematology Association
Jessica Mastrodomenico, *EHA/HemaSphere, The Hague, The Netherlands*

### Editorial Board
Frederic Baron, *University of Liège, GIGA Institute, Liège, Belgium*
Grzegorz Basak, *Medical University of Warsaw, Poland*
Andrea Biondi, *University of Milano-Bicocca, Italy*
Jonathan Bond, *University College Dublin, Ireland*
Dominique Bonnet, *The Francis Crick Institute, London, United Kingdom*
Arndt Borkhardt, *University of Düsseldorf, Germany*
Monika Brüggemann, *University Hospital Schleswig-Holstein, Kiel, Germany*
David Bryder, *Lund University, Sweden*
Ryan Cassaday, *Fred Hutchinson Cancer Center, Seattle, Washington, United States*
Giancarlo Castaman, *Azienda Ospedaliera Universitaria Careggi, Florence, Italy*
Anna Castleton, *The Christie Hospital NHS Foundation Trust, Manchester, United Kingdom*
Nicholas Chiorazzi, *Northwell Health, Manhasset, New York, United States*
Emmanuelle Clappier, *Hôpital Saint-Louis, Paris, France*
Paolo Corradini, *Università degli Studi di Milano, Italy*
Lucia De Franceschi, *University of Verona, Italy*
Kim De Keersmaecker, *University of Leuven, Belgium*
Marianne De Montalembert, *Hôpital Universitaire Necker-Enfants Malades, Paris, France*
Michel Delforge, *Leuven Cancer Institute, Leuven, Belgium*

Catherine Diefenbach, *Perlmutter Cancer Center, New York, United States*
Carlos Fernández De Larrea, *Hospital Clínic Barcelona, Spain*
Francesca Gay, *University of Turin, Italy*
Paolo Ghia, *Università Vita Salute San Raffaele, Milan, Italy*
Domenico Girelli, *Universita degli Studi di Verona, Italy*
Florian Grebien, *University of Veterinary Medicine Vienna, Austria*
Hildegard Greinix, *Medical University of Graz, Austria*
Kirsten Grønbæk, *Rigshospitalet, Copenhagen, Denmark*
Iqbal Hamza, *University of Maryland School of Medicine, Baltimore, Maryland, United States*
Florian Heidel, *University Medical Center Greifswald, Germany*
Olaf Heidenreich, *Princess Máxima Center, Utrecht, The Netherlands*
Brian Huntly, *University of Cambridge, United Kingdom*
Elaine Jaffe, *National Cancer Institute, Bethesda, Maryland, United States*
Mats Jerkeman, *Lund University, Sweden*
Efstathios Kastritis, *National and Kapodistrian University of Athens, Greece*
Léon Kautz, *Inserm, Toulouse, France*
David Kent, *University of York, United Kingdom*
Ulf Klein, *University of Leeds, United Kingdom*
Joost Kluiver, *Universitair Medisch Centrum Groningen, The Netherlands*
Daniela Krause, *University of Frankfurt, Germany*
Nicolaus Kröger, *University Hospital Hamburg-Eppendorf, Germany*
Steven Lane, *QIMR Berghofer Medical Research Institute, Australia*
Frank Leebeek, *Erasmus MC Cancer Institute, Rotterdam, The Netherlands*
Florent Malard, *INSERM, Saint-Antoine Hospital, Paris, France*
Maria-Victoria Mateos, *University of Salamanca, Spain*
Francesco Maura, *Memorial Sloan Kettering Cancer Center, New York, United States*
Simon Mendez-Ferrer, *University of Cambridge, United Kingdom*
Thomas Mercher, *Gustave Roussy Institute, Villejuif, France*
Martina Muckenthaler, *Heidelberg University Hospital, Heidelberg, Germany*
Cesar Nombela Arrieta, *University Hospital Zurich, Switzerland*
James O'Donnell, *Royal College of Surgeons in Ireland, Dublin, Ireland*
Kostas Pantopoulos, *McGill University, Montreal, Quebec, Canada*
Jakob Passweg, *University of Basel, Switzerland*
Heike Pfeifer, *University Hospital Frankfurt, Germany*
Marc Raab, *Heidelberg University Hospital, Heidelberg, Germany*
Marc Raaijmakers, *Erasmus Medical Center Cancer Institute, Rotterdam, The Netherlands*
Alejo Rodriguez-Fratelli, *Institute for Research in Biomedicine, Barcelona, Spain*
Richard Rosenquist, *Karolinska Institutet, Stockholm, Sweden*
Deepa Sampath, *MD Anderson Cancer Center, Houston, Texas, United States*
Rebekka Schneider, *Erasmus MC, Rotterdam, The Netherlands*
Marie Scully, *University College London Hospitals, NHS Foundation Trust, London, United Kingdom*
Kostas Stamatopoulos, *CERTH, Centre for Research and Technology Hellas, Thessaloniki, Greece*
Anna Maria Sureda Balari, *Institut Català d'Oncologia, Barcelona, Spain*
Evangelos Terpos, *National and Kapodistrian University of Athens, Greece*
Alexandre Theocharides, *University Hospital Zürich, Switzerland*
Jan Trka, *University of Prague, Czech Republic*
Karen Vanhoorelbeke, *KU Leuven, Laboratory for Thrombosis Research, Kortrijk, Belgium*
Carlo Visco, *University of Verona, Italy*
Clive Zent, *University of Rochester Medical Center, Rochester, New York, United States*
